# Development of a cloud-based Bioinformatics Training Platform

**DOI:** 10.1093/bib/bbw032

**Published:** 2016-04-15

**Authors:** Jerico Revote, Nathan S Watson-Haigh, Steve Quenette, Blair Bethwaite, Annette McGrath, Catherine A Shang

**Affiliations:** Bioplatforms Australia, Sydney, Australia

**Keywords:** bioinformatics, training, next-generation sequencing, NGS, cloud, workshop

## Abstract

The Bioinformatics Training Platform (BTP) has been developed to provide access to the computational infrastructure required to deliver sophisticated hands-on bioinformatics training courses. The BTP is a cloud-based solution that is in active use for delivering next-generation sequencing training to Australian researchers at geographically dispersed locations. The BTP was built to provide an easy, accessible, consistent and cost-effective approach to delivering workshops at host universities and organizations with a high demand for bioinformatics training but lacking the dedicated bioinformatics training suites required. To support broad uptake of the BTP, the platform has been made compatible with multiple cloud infrastructures. The BTP is an open-source and open-access resource. To date, 20 training workshops have been delivered to over 700 trainees at over 10 venues across Australia using the BTP.

## Introduction

The rapid advance and accessibility of genomic and other ‘omic’ technologies is driving a worldwide demand for bioinformatics training for life scientists. In Australia, to meet this challenge, we have established roadshow-style next-generation sequencing (NGS) bioinformatics hands-on training workshops [1]. The courses are delivered and content maintained by a network of trainers. The major obstacle we initially encountered in delivering bioinformatics training is a shortage of training facilities with the necessary computational specifications and software tools required already installed. In our experience, dedicated computer facilities for bioinformatics training are rare. Generally, courses are run on an *ad hoc* basis, using university computer laboratories during semester break periods where local systems administrators would install software tools and data sets. The more adventurous trainers run workshops using participant’s own laptops, where each participant would need to download and install the necessary software tools ahead of the workshop. This offers no opportunity to verify the training environment, and valuable workshop time can be lost troubleshooting software issues.

To set up a bioinformatics training room from scratch is not straightforward. Logistically, it requires the coordination and support from local IT personnel to oversee the installation of software tools on their systems. More often than not this is further complicated by a lack of suitable computational resources sufficient for analysing data and limited local data storage for the larger analysis and reference data sets. To overcome these issues, we have developed the cloud-based Bioinformatics Training Platform (BTP) to automate the provisioning of computational resources, training materials and software tools on-demand for delivering a 3 day NGS hands-on bioinformatics training workshop.

Before running a bioinformatics workshop, the required number of training virtual machines, one per trainee, are launched using the latest BTP virtual machine image. The creation of the BTP virtual machine images is automated to make it fast, reliable and reproducible. When the training virtual machines are launched from the BTP virtual machine image, they are configured automatically using a collection of orchestration templates, which installs the data sets and training materials for each of the training modules. Local storage issues have been sidestepped by centralizing all test and reference data sets onto cloud object storage. The orchestration process is described in the workflow section of this article. Through the Australian NeCTAR Research Cloud, the BTP has been given a sufficient allocation for running 50 instances with specifications of two CPUs and 8 GB of RAM each.

To maximize the usability of the BTP by our trainer network and others, it has been made available on multiple cloud infrastructure and as a stand-alone virtual machine image. Although the BTP is routinely run using the NeCTAR Research Cloud, it is also available on the Amazon Web Services (AWS). The BTP virtual machine images are freely available on AWS as public Amazon Machine Images (AMI). In addition, we have produced stand-alone virtual machine images of the BTP for the VirtualBox and VMWare desktop virtualization software. These are easily downloadable and runnable on commodity desktop machines. Post workshop, the availability of the BTP provides the opportunity for all workshop trainees to continue working on the tutorials either on the cloud or by using the stand-alone virtual machine images.

The BTP provides bioinformatics training course organizers and trainers an easy solution to remove the set-up and computational resource barriers associated with using basic computer teaching laboratories at universities and other research institutions. The host organization located anywhere in the world is only required to provide Internet access and to install the remote desktop software onto the client machines to enable remote desktop access to the training virtual machines running on the cloud.

In this article, we discuss the process of migrating an entire workshop to the cloud environment, and the components of the BTP that allow this to be automated, and demonstrate how the BTP can be used by giving various workflows examples. It is intended to be a guide for training organizers and system administrators on how the BTP can be deployed on-demand on cloud infrastructures for delivery of hands-on training anywhere. The BTP is an open-source project and is available to be reused and extended by the international bioinformatics training community.

## The BTP components

To deliver a 3 day NGS training workshops reproducibly on multiple cloud infrastructures required the development of a BTP. The first step was to create a framework for the organization and management of the essential components of a workshop such as tutorials, presentations, data sets and tools using a distributed version control system (git). The 3 day NGS workshop has been organized into seven training modules: introduction to the command line, quality control of the NGS data and Alignment, ChIPSeq, RNASeq, *De novo* Assembly and Post Workshop. All the presentations, tools, data sets and tutorials for each training module are accessible and maintained on GitHub (https://github.com/BPA-CSIRO-Workshops). Each training module is represented as a subdirectory within the workshop repository and contains the handouts, data sets, tools and presentations necessary for incorporating individual training modules into a new BTP image. The details of how the framework is used for collaborative development, sharing and reuse are described in our companion paper [2].

**Figure 1.**
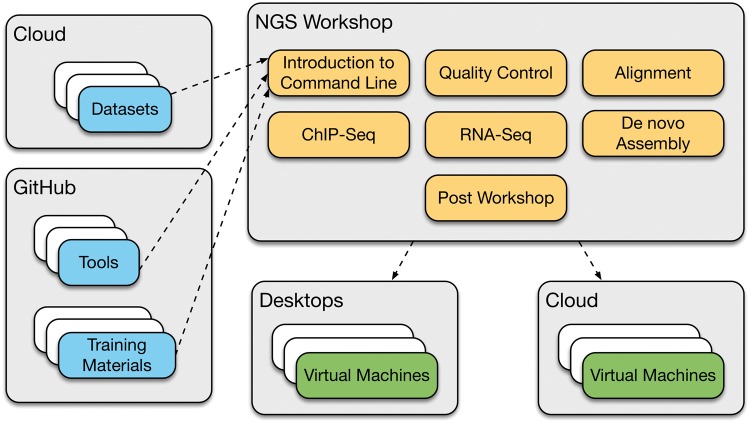
An overview of the architecture and components of the Bioinformatics Training Platform (BTP). A training workshop is composed of various training modules. Each training module is associated to a set of data sets, analysis tools and training materials. Each of the training modules included in the BTP is accessible and maintained on GitHub (https://github.com/BPA-CSIRO-Workshops).

We have leveraged off this framework to set up a system that allows the building of a training image to be automated. The relevant tools, presentations and tutorials are automatically pulled from the GitHub repositories and data sets from the cloud storage and built into the image before launching on the respective environments (Figure 1). Testing is done during the image creation process. Using this approach, the training virtual machines are immediately ready to be used by the trainees. Automating this process is a step-change in the way that training courses are run. To our knowledge, from speaking with other trainers nationally and internationally, training images are usually assembled by hand. This involves the launching of a base image and then all the dependencies, libraries, tools and data sets are added one at a time. This is a labour-intensive task and requires testing of the final product before it can be used for training. Additionally, sharing and reusability of these images are not always straightforward, as the images are only available locally within that computer laboratory environment. In comparison, making the BTP virtual machine images available in the cloud allows it to be easily shared with other users. When a BTP virtual machine image is created on the NeCTAR Research Cloud, it is already available to be launched on all availability zones inside Australia. Similarly, on Amazon Web Services (AWS), BTP AMIs can be easily copied to other AWS regions using a simple interface via the Web console or AWS Command Line Interface (CLI). Without automating the BTP virtual machine image creation process, rerunning popular training workshops would be difficult.

### Orchestration

To automate the building of the BTP images, we have used the Packer software. Packer is an open-source tool that automates the image creation process on a wide variety of platforms, e.g. OpenStack, AWS, VirtualBox and VMWare. Packer uses a simple template written in JavaScript Object Notation (JSON), which is easy to write and maintain. This template contains a builders section, which is responsible for generating machine images. It also has a provisioners section, which defines how to install and configure software tools on the virtual machine images. The set of provisioners included in the orchestration repository contains a mixture of shell scripts and Puppet manifests. Puppet is a configuration management system that installs the software tools on the cloud images (NeCTAR, AWS) and stand-alone images (VirtualBox, VMWare) during virtual machine image creation. It also installs the data sets onto the stand-alone images. When training virtual machines are launched using our orchestration templates, Puppet is executed for installation of the training modules’ data sets.

We have developed Packer templates for creating the cloud images for the NeCTAR Research Cloud and AWS as well as stand-alone images for VirtualBox and VMWare. These Packer templates are found within the orchestration subdirectory of the workshop repository (https://github.com/BPA-CSIRO-Workshops/btp-workshop-ngs.git). The BTP virtual machine image used in the workshops is based on a plain installation of Ubuntu long-term support (LTS) Linux operating system. Managing the Packer templates on GitHub allows them to be accessible and easily maintained. These templates allow the BTP virtual machine images to be easily created, reproduced and reused.

BTP instances can be easily launched on the NeCTAR Research Cloud and AWS using our developed orchestration templates. We have developed orchestration templates that are compatible with NeCTAR Research Cloud’s orchestration service, called Heat, and AWS’s CloudFormation. These templates are also included within the orchestration subdirectory of the workshop repository. The data sets are stored on the cloud object storage and are organized into buckets (also known as containers), one per training module. The data sets used on the BTP are available on both the NeCTAR Research Cloud (Swift) and Amazon S3 object storage (Table 1). The data sets are downloaded on the training virtual machines using Puppet. Puppet reads a simple data set metadata file that describes what data set is required for each training module and its storage location. The data set metadata file is written in YAML (Yet Another Markup Language), a human readable format and easily parsable format. The use of cloud object storage is ideal, as the data sets are mostly static and are easily retrievable using standard Web protocols. An example of a data set metadata file based on the quality control module is described in the supplementary document.

**Table 1.** Training module containers on NeCTAR Swift and Amazon S3. Data sets for each training module are stored on their corresponding containers

**Training module**	**NeCTAR Swift**	**Amazon S3**
Introduction to command line	NGSDataCommandLine	ngs-data-cli
Data quality	NGSDataQC	ngs-data-qc
Read Alignment	NGSDataChIPSeq	ngs-data-chipseq
ChIP-Seq	NGSDataChIPSeq	ngs-data-chipseq
RNA-Seq	NGSDataRNASeq	ngs-data-rnaseq
De novo genome assembly	NGSDataDeNovo	ngs-data-denovo

Specialist bioinformatics software tools for analysis of data are essential for any bioinformatics hands-on workshop. These software tools are packaged and installed into the BTP image on creation. To improve the software tool installation process onto the BTP virtual machine images, we have used a tool packaging system. All the software tools used during the workshop have been packaged into a Debian-based installer. A list of these tools can be found in Table 2.

**Table 2.** Current list of analysis tools included and maintained on the Bioinformatics Training Platform. These tools are automatically configured and installed on the BTP images and instances

**Tool**	**Function**	**Link**
AMOS Hawkeye	Genome data visualization	http://sourceforge.net/projects/amos/
BEDTools	Genome data manipulation	http://bedtools.readthedocs.org/en/latest/
BLAT	Sequence location lookup in the genome	https://genome.ucsc.edu/FAQ/FAQblat.html
Bowtie	Read Alignment	http://bowtie-bio.sourceforge.net/index.shtml
CummeRbund	RNA-Seq analysis using R	http://bioconductor.org/packages/release/bioc/html/cummeRbund.html
Cufflinks	RNA-Seq analysis	http://cole-trapnell-lab.github.io/cufflinks/
DESeq2	Differential gene expression analysis using R	https://bioconductor.org/packages/release/bioc/html/DESeq2.html
edgeR	Empirical gene expression analysis using R	https://bioconductor.org/packages/release/bioc/html/edgeR.html
FastQC	FastQC	http://www.bioinformatics.babraham.ac.uk/projects/fastqc/
FASTX	Toolkit for short reads preprocessing	http://hannonlab.cshl.edu/fastx_toolkit/
IGV	Interactive exploration of genomic data	https://www.broadinstitute.org/igv/
igvtools	For preprocessing data before loading to IGV	https://www.broadinstitute.org/igv/igvtools
MACS	ChIP-Seq analysis	http://liulab.dfci.harvard.edu/MACS/
MUMmer	Rapid genome alignment, a dependency for AMOS	http://mummer.sourceforge.net/
PeakAnalyzer	Multi-peak data analysis	http://www.bioinformatics.org/peakanalyzer/wiki/
Picard	Sequence data analysis	http://broadinstitute.github.io/picard/
SAMtools	For manipulating alignments in the SAM format	http://samtools.sourceforge.net/
Skewer	Adapter trimmer for paired-end reads	https://github.com/relipmoc/skewer

To package the software tools, we have used the FPM Cookery software, which then uses the Effing Package Management (FPM) package builder. FPM Cookery uses tool recipes to build the software tools and produce the Debian-based installer. The tool recipes are Ruby-based code that contain instructions on how a specific software tool is built. This includes getting the software tool’s artefact, normally the source code, then instructions on how to configure and compile the software tool, and finally, on how to install the software tool’s executable into the operating system. The FPM Cookery tool recipes are managed in GitHub (https://github.com/BPA-CSIRO-Workshops/btp-tools.git). The tool recipe repository is integrated with the Travis CI service. Travis CI is an open-source continuous integration system used for automatic building and testing of projects hosted on repository hosting services such as GitHub. When a software tool recipe is updated or a new software tool recipe is added to the repository, it will automatically submit new builds to the Travis CI service as part of the continuous integration process. Travis CI will automatically send notifications to report the build status. An example of a tool recipe for packaging the Bowtie2 aligner tool is described in the supplementary document.

**Figure 2.**
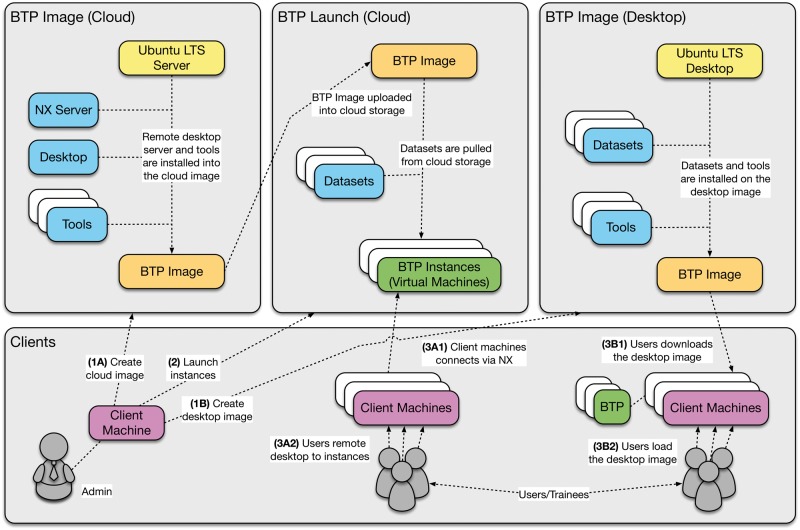
An overview of the major BTP workflows. The main workflows are as follows: (1) deployment of the BTP on the cloud and desktop environments and (2) creation of the BTP images. These workflows allow reuse and extension of the BTP for delivering hands-on training workshops.

Various workflows using the BTP for developing workshops can be found in GitHub (https://github.com/BPA-CSIRO-Workshops/btp-workshop-template) along with detailed README documentation to assist users. The workshop information can also be accessed from a landing page (http://bpa-csiro-workshops.github.io/) containing information about the workshops and links on how to get started. Workflows demonstrating how the BTP can be deployed using different environments for training workshops are described in the workflows section below, and an overview is shown in Figure 2.

## BTP Workflows

### Use Case 1. Reusing an existing BTP virtual machine image for running a NGS training workshop

The BTP can be easily reused by other trainers to run the NGS course described in this article. Below, we provide an overview of the three workflows for running this course on various platforms: (i) using AWS, (ii) using NeCTAR Research Cloud and (iii) using VirtualBox or VMWare with stand-alone virtual machine images. Additional detailed information is available at the BTP orchestration page in GitHub (https://github.com/BPA-CSIRO-Workshops/btp-orchestration).

#### i. Launching BTP virtual machines on AWS

AWS users can search for the latest BTP virtual machine image (AMI) from the available public images (Table 3) and launch a fully configured training instance using AWS CloudFormation. An orchestration template (cloudformation/ngs.json) for deploying the BTP on AWS is included in the workshop repository (https://github.com/BPA-CSIRO-Workshops/btp-orchestration). This orchestration template pulls down the relevant training module data sets from Amazon S3 into the launched training virtual machines.

**Table 3.** Versions and location of the latest BTP virtual machine images for the cloud and desktops. The virtual machine images are publicly available and accessible from the NeCTAR Research Cloud and AWS. The stand-alone virtual machine images are downloadable from the workshop releases page on GitHub

**Environment**	**Image information**
NeCTAR	Name: BTP-2016-01-25, ID: 9c7fd8ac-5cfe-4067-aae3-82e53a49def3
AWS	Region: ap-southeast-2, AMI: ami-be597dddRegion: eu-west-1, AMI: ami-d3a31ea0
VirtualBox	https://github.com/BPA-CSIRO-Workshops/btp-workshop-ngs/releases
VMWare	https://github.com/BPA-CSIRO-Workshops/btp-workshop-ngs/releases

#### ii. Launching BTP virtual machines on the NeCTAR Research Cloud

The BTP virtual machine images are publicly available on the NeCTAR Research Cloud. The image names to search for are documented in Table 3. The training manual and the data sets are installed on the training virtual machine as a post-launch step using Puppet. An orchestration template (https://github.com/BPA-CSIRO-Workshops/btp-orchestration) for launching and configuring training virtual machines on the NeCTAR Research Cloud has been developed to help users and provide an easy and fast access to the BTP.

#### iii. Launching BTP virtual machines on VirtualBox and VMWare using the stand-alone virtual machine images

All the BTP components (data sets, analysis tools and training manual) are packaged up together into the stand-alone virtual machine images available for VirtualBox and VMWare. Users can easily download (Table 3) the latest virtual machine images and import it directly to their chosen desktop virtualization software. The required performance and integration packages for both VirtualBox and VMWare are built-in into the virtual machine images to ensure seamless graphical operation. Once the virtual machine image has been imported into the desktop virtualization software, it can be immediately started up. After the BTP virtual machine has finished booting up, the standard login screen will be presented. The built-in user (ubuntu) has been setup with admin privileges so that users can perform system updates and upgrades on the virtual machine easily.

### Use Case 2: Rebuilding a NGS virtual machine image before running a new workshop

To incorporate changes such as updates to the underlying operating system, tools and data sets, the BTP virtual machine image is recreated to ensure it is up to date and ready for a new workshop to be delivered. This process varies slightly depending on the computing environment chosen for delivering the workshop but generally takes 1 day to complete. Below, we outline the following three workflows: (i) creating a new virtual machine image for AWS; (ii) creating a new virtual machine image for the NeCTAR cloud; (iii) creating a new virtual machine image for VirtualBox and VMWare. A step-by-step procedure is contained within the README documentation found in the BTP Orchestration repository (https://github.com/BPA-CSIRO-Workshops/btp-orchestration).

The current BTP virtual machine image is built from a vanilla (base) installation of the Ubuntu LTS using the Packer software. The Packer software uses the Packer template to orchestrate the commands that direct the process of creating an image. Although Packer software makes creating machine images easier and faster through orchestration, it does not allow you to manage the configuration of the BTP virtual machine image. For this, we have used Puppet to install the analysis tools and data sets onto the Packer-made images. The configuration file used to define what image we want to build is called a template in Packer terminology and is written in JSON, a human-readable format. The configuration files for creating images for the NeCTAR Research Cloud, AWS, VirtualBox and VMWare all follow a similar structure.

#### i. Creating a new virtual machine image for Amazon Web Services (AWS)

A Packer template for building the BTP virtual machine images as AMI is included in the BTP orchestration repository (https://github.com/BPA-CSIRO-Workshops/btp-orchestration). Users will use their AWS credentials to provision Elastic Compute Cloud (EC2), Elastic Block Storage (EBS) and S3 resources for the automated creation of the BTP virtual machine image. Users can trigger the build process using the Packer software on their client machines. The overall build process on AWS is similar to the NeCTAR Research Cloud in regards to the installation of the base operating system and remote desktop server. Specific AWS metadata is included in the Packer template. The build process will launch an EBS-backed EC2 instance on AWS based on the public Ubuntu AMI. Packer will then load individual provisioners into this instance to configure it for the training platform. The resulting virtual machine image is then published as a public AMI on AWS (Table 3).

#### ii. Creating a new virtual machine image for the NeCTAR Research Cloud

A simple Packer template containing the virtual machine metadata and build instructions is used in conjunction with the Packer software to create the BTP NeCTAR virtual machine image. This configuration file can be found in the BTP orchestration repository (https://github.com/BPA-CSIRO-Workshops/btp-orchestration). A user installs the Packer software on a local Linux-based machine, and then clones the workshop repository from the GitHub project. Using the Packer command-line interface, the image building process can be triggered with the template as its input. The entire process usually takes several minutes to an hour to complete, and once done, produces the BTP virtual machine image (Table 3). The generated virtual machine image is in a QEMU Copy on Write file format that is compatible with the NeCTAR Research Cloud. Using the OpenStack command-line clients and the users OpenStack credentials, the BTP virtual machine image is then uploaded and made public on the NeCTAR image catalogue. The build process can be rerun and the Packer template customized if required. This is useful for users who want to deploy their own version of the virtual machine image, with updated tools.

#### iii. Creating a new virtual machine image for VirtualBox and VMWare

Similar to how virtual machine images are created for the NeCTAR Research Cloud and the AWS, creating new BTP virtual machine images that can be imported into the VirtualBox and VMWare virtualization platforms are done using the Packer software. The BTP orchestration repository (https://github.com/BPA-CSIRO-Workshops/btp-orchestration) contains the Packer template for both VirtualBox and VMware. The template contains parameters for creating the BTP virtual machine image based on the latest Ubuntu LTS ISO image available. After a successful build, the corresponding virtual machine images will be saved into the output directories specified in the Packer template. These virtual machine images can then be distributed and made available online by uploading it onto a public container on the cloud.

### Use case 3: Customizing the NGS virtual machine image

A trainer would like to run an existing BTP-NGS workshop; however, owing to time constraints or specific workshop requirements, changes need to be made to the images, new modules added or removed, for example. The BTP makes this straightforward. For example, new training modules can be added or truncated using the training module template file managed on the BPA-CSIRO GitHub project. The orchestration system and the handout build mechanism will then automatically detect what training modules are included in the current environment, providing a ‘plug and play’ model, which promotes reusability and collaboration [2].

#### i. Adding New Tools and Data *sets to the BTP*

To add new tools and data sets, the relevant metadata files need to be modified. The tools and data set metadata files are included for each training modules inside the workshop. For addition of new tools, the tools metadata file must be updated with the new tool’s installer location. Additionally, new data sets can be added to a training module easily if they are accessible using a public URL. This can be achieved, for example, by uploading the new data sets to object storage and making it publicly readable. The new data set file names, their location on the Web and their destination path on the BTP virtual machine images can then be added into the data set metadata file. The Puppet configuration system will then read this new information from the metadata files and install the new tool or data set onto the BTP virtual machine image.

## Discussion

Cloud computing has made it possible to deliver hands-on bioinformatics training courses on-demand around Australia (Table 4). This has enabled the training to have a much wider outreach than would be possible if training courses were delivered in a single location. It is now possible to easily run workshops in regional areas with only basic computer hardware as long as there is a decent connection to the Internet.

**Table 4.** Previous training workshops conducted using the Bioinformatics Training Platform. The training workshops were conducted at various institutions and locations across Australia

**Year**	**Workshop name**	**Start date**	**End date**	**Location**	**Host institute**	**No. of Attendees**
2012	Bioplatforms Australia and CSIRO joint 2-Day Introduction to NGS hands-on workshop	12/07/2012	13/07/2012	Melbourne	Monash University	22
2012	Bioplatforms Australia and CSIRO joint 2-Day Introduction to NGS hands-on workshop	16/07/2012	17/07/2012	Sydney	University of New South Wales	22
2012	Bioplatforms Australia and CSIRO joint 2-Day Introduction to NGS hands-on workshop	13/11/2012	14/11/2012	Brisbane	University of Queensland	33
2012	Bioplatforms Australia and CSIRO joint 2-Day Introduction to NGS hands-on workshop	15/11/2012	16/11/2012	Adelaide	University of Adelaide	29
2013	Bioplatforms Australia and CSIRO joint 3-Day Introduction to NGS hands-on workshop	11/02/2013	13/02/2013	Canberra	CSIRO	35
2013	Bioplatforms Australia and CSIRO joint 3-Day Introduction to NGS hands-on workshop	19/06/2013	21/06/2013	Perth	Curtin University	38
2013	Bioplatforms Australia, CSIRO and EMBL joint 1-Day Introduction to NGS quality and alignment hands-on workshop for the EMBL-Aus PhD program	09/07/2013	09/07/2013	Melbourne	Monash University	60
2013	Bioplatforms Australia, CSIRO and EMBL joint 3-Day Introduction to NGS hands-on workshop	10/07/2013	12/07/2013	Melbourne	Monash University	38
2013	Bioplatforms Australia, CSIRO and EMBL joint 3-Day Introduction to NGS hands-on workshop	20/11/2013	22/11/2013	Sydney	University of New South Wales	38
2013	Bioplatforms Australia, CSIRO and EMBL joint 3-Day Introduction to NGS hands-on workshop	25/11/2013	27/11/2013	Brisbane	University of Queensland	30
2014	Bioplatforms Australia, CSIRO and EMBL joint 2-Day Introduction to Metagenomics hands-on workshop	06/02/2014	07/02/2014	Sydney	University of New South Wales	38
2014	Bioplatforms Australia, CSIRO and EMBL joint 2-Day Introduction to Metagenomics hands-on workshop	10/02/2014	11/02/2014	Melbourne	Monash University	34
2014	Bioplatforms Australia, CSIRO and EMBL joint 3-Day Introduction to NGS hands-on workshop	01/07/2014	03/07/2014	Sydney	University of New South Wales	37
2014	Bioplatforms Australia, CSIRO and EMBL joint one-Day Introduction to NGS quality control and alignment hands-on workshop for the EMBL-AUS PhD program	08/07/2014	08/07/2014	Canberra	Australian National University	60
2014	Bioplatforms Australia, CSIRO and EMBL joint 2-Day Introduction to Metagenomics hands-on workshop	09/07/2014	10/07/2014	Canberra	Australian National University	15
2014	Bioplatforms Australia, CSIRO and EMBL joint 3-Day Introduction to NGS hands-on workshop	10/11/2014	12/11/2014	Sydney	University of Sydney	13
2014	Bioplatforms Australia, CSIRO and EMBL joint 3-Day Introduction to NGS hands-on workshop	25/11/2014	27/11/2014	Hobart	University of Tasmania	20
2014	Bioplatforms Australia, CSIRO and EMBL joint 2-Day Introduction to Metagenomics hands-on workshop	10/12/2014	11/12/2014	Townsville	James Cook University	42
2015	Bioplatforms Australia, CSIRO and EMBL joint 3-Day Introduction to NGS hands-on workshop	06/23/15	06/25/15	Brisbane	Queensland University of Technology	43
2015	Bioplatforms Australia, CSIRO and EMBL joint 1-Day Introduction to NGS quality and alignment hands-on workshop for the EMBL-Aus PhD program	06/26/15	06/26/15	Perth	University of Western Australia	60

Importantly, for anyone involved in providing hands-on training courses, using cloud infrastructure is a great way to reduce running costs. All the upfront costs of providing and maintaining large numbers of high specification hardware are removed. In addition, the time, effort and expertise required by systems administrators to set up the computational infrastructure for each workshop in different venues is greatly reduced, as the workshops are managed centrally by a single system administrator. Generally, it takes around 1 day to rebuild the NGS virtual machine image before a workshop and a couple of hours to launch 40 or so virtual machines images. Moreover, the trainees benefit from having a consistent training environment that is accessible after the workshop. The trainers also benefit, as all tools have been installed and are working ahead of the workshop, minimizing the amount of time and effort spent on troubleshooting issues on individual computers.

Australian-based researchers are automatically given 2 cores worth of computational resources on the NeCTAR Research Cloud. It is possible to apply to NeCTAR for a free bigger allocation for training purposes. For example, we currently have a NeCTAR allocation that can accommodate 50 BTP instances (2 cores, 8 GB of memory) with 60 GB of storage per instance. The BTP can now also be used on AWS to deliver hands-on training workshops. AWS provides free tier resources to users to get started easily on Amazon EC2. In our experience, a 3 day workshop consumes ∼10 instance hours on Amazon EC2 per instance per day using the ‘m1.large’ Amazon EC2 instance type. Current AWS cloud pricing is available on the AWS website.

Out of necessity and with the long-term aim to maximize the number of workshops that we can deliver using a volunteer workforce of developers and trainers, we have automated the management of our infrastructure platform to greatly increase the speed of deployment and reuse. This investment in automation and orchestration of our virtual machine images means that setting up of >40 virtual machines for each new workshop does not rely on tedious and repetitive manual builds and provisioning. Importantly, this allows changes to be made and implemented quickly by simply modifying the relevant orchestration template. The use of orchestration templates allows us to easily update and build new virtual machine images for every new workshop. This approach ensures the images are reliable and reproducible, as each new build is tested and bugs can be detected in advance and will not cause havoc in the middle of a workshop.

As previously mentioned, there are several ways that workshops are currently being delivered (local Virtual Machines as well as installing tools and data sets manual on desktops), and advantages and disadvantages in using these have been highlighted above. Another mode of delivery model is the bring your own device approach used by Software Carpentry (SWC) [3]. SWC teaches students best practices in computing skills such as Python and R using a network of distributed trainers worldwide. The Bring Your Own (BYO) device workshop model is ideal for teaching programming languages, as they require only a text editor and the Unix environment. This means there is little to prepare in advance. In contrast, the domain-specific bioinformatics training courses that we run need a specific operating system, specific tools installed, access to large data sets and computational power for analysis. Adopting a BYO model for workshops run using the BTP is technically possible now that the BTP is in place. The trainee could install a client on their own device to access the training virtual images. This approach will be piloted in upcoming workshops.

The BTP was designed to provide a lean, minimal training environment, which contains only the relevant tools and data sets required for a specific workshop. As such, it is more flexible and easier to maintain compared with other Linux platforms CloudBioLinux [4] and Galaxy [5], which are aimed at production bioinformatics. However, if deployed with sufficient resources, a trainee could use the BTP to analyse his/her own larger data sets using the same tools and approaches learned during the workshops.

### Future developments for the BTP

To improve the distribution of the BTP on Linux machines, we are currently testing Docker. Docker is a software container technology that has gained popularity and wide user adoption since its first release in 2013. The Docker image will have all the instructions for installing software. Docker provides an API for automating rapid deployments of applications on lightweight containers using shared operating systems. Docker enables application portability across diverse environments, seamless version control and sharing. We aim to make the BTP available as Docker images, which can be easily tested, deployed and reused on various environments running Docker. We are also working on making the BTP public AMI available on other AWS regions.

The development of the BTP is a large collaborative effort, involving multiple organizations both nationally and internationally and was initiated in 2012 to overcome a major problem of how to provide hands-on bioinformatics training, regardless of location or limited computer hardware resources across Australia. There is now a community of Australian trainers using the BTP. The BTP is freely available to anyone who wants to run a bioinformatics training course on the cloud. We encourage bioinformatics training communities to use this infrastructure and welcome workshop contributions to this community resource.

## Supplementary Data

Supplementary data are available online at http://bib.oxfordjournals.org/.

## Supplementary Material

Supplementary Data

## Notes

**Jerico Revote** is a software developer at the Monash eResearch Centre involved in research collaborations and R@CMon—the Research Cloud at Monash University.

## Notes

**Nathan S.Watson-Haigh** is a bioinformatician with expertise and interest in wheat genomics and transcriptomics, systems biology approaches, de novo genome assembly, next-generation sequencing data analysis, scientific computing infrastructure, cloud computing and workshop development and delivery.

## Notes

**Steve Quenette** is the deputy director of the Monash eResearch Centre and project sponsor of the NeCTAR Research Cloud node at Monash University, with expertise in research platforms and productivity, and a research interest in computational science.

## Notes

**Blair Bethwaite** is a Senior High Performance Computing (HPC) Consultant at the Monash Consultant at the Monash eResearch Centre, and he is the technical architect and lead for R@CMon—the Research Cloud at Monash University and a node of the NeCTAR Research Cloud. He has interest in the scientific applications of high-throughput computing.

## Notes

**Annette McGrath** is the Bioinformatics Core Leader at the Commonwealth Scientific and Industrial Research Organisation. She leads a team of bioinformaticians involved in genomics research, bioinformatics infrastructure and tool development and provision and with a strong interest in training and development.

## Notes

**Catherine A. Shang** is a project manager at Bioplatforms Australia, managing Australian collaborative research consortiums. Catherine is actively involved in establishing bioinformatics communities and developing and delivering open-access bioinformatics training courses for the benefit of the Australian research community.
