# Supplementary material for: Development of a cloud-based Bioinformatics Training Platform
Source: Brief Bioinform. 2016 Apr 15;18(3):537–44. doi: 10.1093/bib/bbw032 (PMC5429016; doi:10.1093/bib/bbw032)
Supplement: Supplementary Data [file bbw032_supp.zip › Table-9.doc]

Table 9: A glossary of terms used in this paper.

| Term | Definition |
| --- | --- |
| AMI | Amazon Machine Image, a cloud image used to launch instances on AWS |
| API | Application Programming Interface |
| AWS | Amazon Web Services |
| BTP | The Bioinformatics Training Platform |
| CLI | Comand Line Interface |
| CloudFormation | AWS’ service for creating and managing AWS resources |
| CSIRO | The Commonwealth Scientific and Industrial Research Organisation |
| Docker | Open-source project for automating deployment of applications using software containers |
| EC2 | Amazon Web Services’s Elastic Cloud Computing |
| EBS | Elastic Block Storage, Amazon's persistent block storage service |
| FPM | Effing Package Manager, used to package tools into stand-alone installers |
| GitHub | A widely popular Web-based Git repository hosting service, used by many open source projects |
| Instance Type | The size and configuration of the virtual machine in terms of CPUs, memory and storage |
| JSON | JavaSript Object Notation, open standard format used for transmitting data with attribute-value pairs |
| LTS | Long-term support, a version of the Linux operating system designed to be supported for longer than normal period |
| NeCTAR | The Australian National eResearch Collaboration Tools and Resources project |
| NGS | Next-Generation Sequencing |
| Object Storage | A storage architecture where data are stored and managed as objects. It is not a traditional file system |
| OpenStack | The open-source cloud operating system used by the NeCTAR Research Cloud |
| Orchestration | The process of automating the deployment and configuration of virtual machine images and instances |
| Packer | A tool for creating identical virtual machine images for various environments |
| Provisioners | Used by the Packer software for installing and configuring software when generating machine images |
| Puppet | Configuration management utility for automated configuration of UNIX-like and Windows environments |
| QCOW | QEMU Copy On Write, a disk image file format used by QEMU |
| QEMU | Short for Quick Emulator, an open-source hosted hypervisor used for virtualization |
| S3 | Simple Storage Service, the AWS object storage service where the BTP datasets are also available |
| Security Keys | Security keys are used for remotely accessing a cloud instance using secure shell (SSH) |
| Security Groups | A security group groups network filter rules that are applied to an instance's networking |
| Swift | The NeCTAR Research Cloud object storage service based on OpenStack |
| Travis CI | Hosted distributed continuous integration system, used for automated building and testing of projects |
| Ubuntu | A Debian-based Linux operating system used in personal computers and servers |
| VirtualBox | A popular software for hosting virtual machines on desktops |
| VMWare | Commercial software for hosting virtual machines on desktops and servers |
| YAML | YAML Ain't Markup Language, a human readable data serialization format used for defining data mappings |
