# Supplementary material for: Development of a cloud-based Bioinformatics Training Platform
Source: Brief Bioinform. 2016 Apr 15;18(3):537–44. doi: 10.1093/bib/bbw032 (PMC5429016; doi:10.1093/bib/bbw032)
Supplement: Supplementary Data [file bbw032_supp.zip › Development of a Cloud-based Bioinformatics Training Platform (Supplementary).doc]

# Development of a Cloud-based Bioinformatics Training Platform (Supplementary)

Jerico Revote, Nathan S. Watson-Haigh, Steve Quenette, Blair Bethwaite, Annette McGrath, Catherine A. Shang

**Jerico Revote** is a software developer at the Monash eResearch Centre involved in research collaborations and R@CMon – the Research Cloud at Monash University.

**Nathan S.Watson-Haigh** is a bioinformatician with expertise and interest in systems biology approaches, de novo genome assembly, NGS data analysis, cloud computing and workshop development and delivery.

**Steve Quenette** is the deputy director of the Monash eResearch Centre and project sponsor of the NeCTAR Research Cloud node at Monash University, with expertise in research platforms and productivity, and a research interest in computational science.

**Blair Bethwaite** is a Senior HPC Consultant at the Monash eResearch Centre, he is the technical architect and lead for R@CMon - the Research Cloud at Monash University and a Node of the NeCTAR Research Cloud. He has a background and research interest in the science applications of high-throughput computing.

**Annette McGrath** is the Bioinformatics Core Leader at the Commonwealth Scientific and Industrial Research Organisation. She leads a team of bioinformaticians involved in genomics research, bioinformatics infrastructure and tool development and provision and with a strong interest in training and development.

**Catherine A. Shang** is a project manager working within large Australian collaborative systems biology research consortiums. Catherine is actively involved in establishing collaborative bioinformatics networks and developing and delivering open access bioinformatics training courses for the benefit of the Australian research community.

# Tools Metadata File

<< Table 5 >>

The tools metadata is written in a hierarchical order (hieradata) and read by Puppet for installing the individual analysis tools onto the BTP images and instances for each of the training modules. The actual filename of the tool installer is specified (Line 2) on the metadata file. It is followed by the URL (Line 3) where the tool installer will be downloaded from. Then it tells (Line 4) the configuration software Puppet to install the tool using the given provider. The same block declaration is done for rest of the analysis tools to be installed.

# FPM Recipe

<< Table 6 >>

The first section (Line 2 to Line 8) of the FPM recipe (Table 6) contains some standard information about the tool being packaged: the tool name, a short description, the tool version, and the location of the tool artifact. In the example tool recipe shown on Table 1, the tool artifact is the source code tar ball location of Bowtie2. Before Bowtie2 is built, we also need to install the build dependencies (Line 11). The next section (Line 14 to Line 18) defines the build process block. The build block instructs FPM Cookery to change the working directory (Line 15) to the extracted Bowtie2 directory, then it executes the “make” command to build the Bowtie2 executables. The last section (Line 21 to Line 25), is the install block. This block tells FPM Cookery to go (Line 22) to the build directory, then it collects the Bowtie2 executables to be included in the Bowtie2 installer package.

After FPM Cookery is finished executing the tool recipe, the installer package, installable on the training images and instances is created. The installer package can then be made available, for example by uploading it to object storage. Once uploaded, the location of the installer package can then be used on the tools metadata on each training module as shown on Table 1. To support reusability and ensure that the tool recipes are always up to date, we’ve integrated the BTP tools repository (https://github.com/BPA-CSIRO-Workshops/btp-tools.git) with the Travis CI system.

# Dataset Metadata File

<< Table 7 >>

The dataset metadata file (Table 7) is also written in a hierarchical order. On line 6, it displays the actual filename of the dataset that will be downloaded and installed on the BTP images and instances. Line 7 specifies the download URL of the object storage container where the dataset is stored. Lines 8 and 9 specifies where on the BTP image and instances the downloaded datasets will be installed. This block is repeated for each dataset used by the training module. This file is read by the Puppet configuration tool for automated installation of the training modules’ datasets.

# Packer Recipe

<< Table 8 >>

The Packer recipe file (Table 8) is used to create the BTP images for the various environments. The recipe file starts first with a simple description (Line 2). This is followed by the builders section, which includes the source URL where the base Ubuntu LTS ISO will be downloaded from. The URL specification is followed by the boot command (Line 20) for auto-prompt booting of the ISO and a set of execution commands for further customization of the virtual machine image. The Packer recipe adjusts (Line 32) the number of vCPUs (virtual cpus), memory and storage devices to be compatible with a commodity computer. The training images are configured to utilise 2 vCPUs, 4-8 gigabytes of RAM and ~30GB for storage. These settings can be configured as per required depending on the target environments. The next section, provisioners (Line 38), contains a set of instructions for the installation of the required system packages and libraries, analysis tools, virtualisation performance packages (VirtualBox Guest Additions, VMWare Tools), and deployment of the training modules and datasets into the virtual machine image. After launching the base training image on the target environment, we use Puppet to configure the BTP. Puppet installs the analysis tools, datasets and training materials onto the virtual machine image.

# Table of Terms

<< Table 9 >>
