# Supplementary material for: Development of a cloud-based Bioinformatics Training Platform
Source: Brief Bioinform. 2016 Apr 15;18(3):537–44. doi: 10.1093/bib/bbw032 (PMC5429016; doi:10.1093/bib/bbw032)
Supplement: Supplementary Data [file bbw032_supp.zip › Table-5.doc]

Table 5: A tools metadata file used by the orchestration system for the installation of the software tools required for the NGS Quality Control module.

| Line | Tools Metadata File |
| --- | --- |
| 1 | btp::analysis_tools: |
| 2 | fastqc_0.11.2-0_amd64.deb: |
| 3 | source: 'https://swift.rc.nectar.org.au:8888/v1/AUTH_809/Tools' |
| 4 | provider: 'dpkg' |
| 5 | fastxtoolkit_0.0.14-0_amd64.deb: |
| 6 | source: 'https://swift.rc.nectar.org.au:8888/v1/AUTH_809/Tools' |
| 7 | provider: 'dpkg' |
| 8 | picard_1.119-0_amd64.deb: |
| 9 | source: 'https://swift.rc.nectar.org.au:8888/v1/AUTH_809/Tools' |
| 10 | provider: 'dpkg' |
| 11 | skewer_0.0.1-0_amd64.deb: |
| 12 | source: 'https://swift.rc.nectar.org.au:8888/v1/AUTH_809/Tools' |
| 13 | provider: 'dpkg' |
