# Supplementary material for: Development of a cloud-based Bioinformatics Training Platform
Source: Brief Bioinform. 2016 Apr 15;18(3):537–44. doi: 10.1093/bib/bbw032 (PMC5429016; doi:10.1093/bib/bbw032)
Supplement: Supplementary Data [file bbw032_supp.zip › Table-6.doc]

Table 6: FPM recipe for the Bowtie2 tool. This recipe is used by FPM Cookery to build, configure and package up Bowtie2 into an Ubuntu package installer.

| Line | FPM Recipe |
| --- | --- |
| 1 | class Bowtie2 < FPM::Cookery::Recipe |
| 2 | description 'Bowtie 2 is an ultrafast and memory-efficient tool for aligning sequencing reads to long reference sequences.' |
| 3 | name 'bowtie2' |
| 4 | version '2.2.4' |
| 5 | revision 0 |
| 6 | homepage 'http://bowtie-bio.sourceforge.net/index.shtml' |
| 7 | source "http://sourceforge.net/projects/bowtie-bio/files/bowtie2/#{version}/bowtie2-#{version}-source.zip" |
| 8 | md5 'd31294875092e76ebb061eadc7998582' |
| 9 |  |
| 10 | # Let's install build dependencies first. |
| 11 | build_depends ['curl', 'g++', 'unzip', 'wget'] |
| 12 |  |
| 13 | # Build: |
| 14 | def build |
| 15 | Dir.chdir "bowtie2-#{version}" do |
| 16 | make |
| 17 | end |
| 18 | end |
| 19 |  |
| 20 | # Install: |
| 21 | def install |
| 22 | Dir.chdir "bowtie2-#{version}" do |
| 23 | bin.install ['bowtie2', 'bowtie2-align-s', 'bowtie2-align-l', 'bowtie2-build', 'bowtie2-build-s', 'bowtie2-build-l', 'bowtie2-inspect', 'bowtie2-inspect-s', 'bowtie2-inspect-l'] |
| 24 | end |
| 25 | end |
| 26 | end |
