# Supplementary material for: Development of a cloud-based Bioinformatics Training Platform
Source: Brief Bioinform. 2016 Apr 15;18(3):537–44. doi: 10.1093/bib/bbw032 (PMC5429016; doi:10.1093/bib/bbw032)
Supplement: Supplementary Data [file bbw032_supp.zip › Table-7.doc]

Table 7: A dataset metadata file used by the orchestration system to install required training datasets into the training images and instances. All data sets are stored centrally on object storage and the metadata file contains all the information to allow the datasets to be automatically sourced and installed on a new virtual machine image.

| Line | Datasets Metadata File |
| --- | --- |
| 1 | btp::modules: |
| 2 | qc: |
| 3 | dirs: |
| 4 | - 'qc' |
| 5 | data: |
| 6 | good_example.fastq: |
| 7 | source: 'https://swift.rc.nectar.org.au:8888/v1/AUTH_809/NGSDataQC' |
| 8 | targets: |
| 9 | - 'qc/good_example.fastq' |
| 10 | adaptorQC.fastq.gz: |
| 11 | source: 'https://swift.rc.nectar.org.au:8888/v1/AUTH_809/NGSDataQC' |
| 12 | targets: |
| 13 | - 'qc/adaptorQC.fastq.gz' |
| 14 | bad_example.fastq: |
| 15 | source: 'https://swift.rc.nectar.org.au:8888/v1/AUTH_809/NGSDataQC' |
| 16 | targets: |
| 17 | - 'qc/bad_example.fastq' |
| 18 | bad_example.fastq.trimmed_fastqc.zip: |
| 19 | source: 'https://swift.rc.nectar.org.au:8888/v1/AUTH_809/NGSDataQC' |
| 20 | targets: |
| 21 | - 'qc/bad_example.fastq.trimmed_fastqc.zip' |
| 22 | qcdemo_R1.fastq.gz: |
| 23 | source: 'https://swift.rc.nectar.org.au:8888/v1/AUTH_809/NGSDataQC' |
| 24 | targets: |
| 25 | - 'qc/qcdemo_R1.fastq.gz' |
| 26 | qcdemo_R2.fastq.gz: |
| 27 | source: 'https://swift.rc.nectar.org.au:8888/v1/AUTH_809/NGSDataQC' |
| 28 | targets: |
| 29 | - 'qc/qcdemo_R2.fastq.gz' |
