# Supplementary material for: Development of a cloud-based Bioinformatics Training Platform
Source: Brief Bioinform. 2016 Apr 15;18(3):537–44. doi: 10.1093/bib/bbw032 (PMC5429016; doi:10.1093/bib/bbw032)
Supplement: Supplementary Data [file bbw032_supp.zip › Table-8.doc]

Table 8: A Packer recipe for building the BTP VirtualBox image.

| Line | Packer Recipe |
| --- | --- |
| 1 | { |
| 2 | "description": "BTP Image Deployment for VirtualBox", |
| 3 | "builders": [ |
| 4 | { |
| 5 | "type": "virtualbox-iso", |
| 6 | "guest_os_type": "Ubuntu_64", |
| 7 | "vm_name": "btp", |
| 8 | "http_directory": "precise", |
| 9 | "iso_url": "http://releases.ubuntu.com/12.04/ubuntu-12.04.5-alternate-amd64.iso", |
| 10 | "iso_checksum": "fa0893ffe2188a6daf918791cdccb480", |
| 11 | "iso_checksum_type": "md5", |
| 12 | "ssh_username": "ubuntu", |
| 13 | "ssh_password": "ubuntu", |
| 14 | "ssh_port": "22", |
| 15 | "ssh_wait_timeout": "60m", |
| 16 | "format": "ovf", |
| 17 | "disk_size": "30000", |
| 18 | "shutdown_command": "echo 'ubuntu' \| sudo -S shutdown -P now", |
| 19 | "headless": "false", |
| 20 | "boot_command": [ |
| 21 | "<esc><esc><enter><wait>", |
| 22 | "/install/vmlinuz noapic ", |
| 23 | "preseed/url=http://{{ .HTTPIP }}:{{ .HTTPPort }}/preseed.cfg ", |
| 24 | "debian-installer=en_US auto locale=en_US kbd-chooser/method=us ", |
| 25 | "hostname={{ .Name }} ", |
| 26 | "fb=false debconf/frontend=noninteractive ", |
| 27 | "keyboard-configuration/modelcode=SKIP keyboard-configuration/layout=USA ", |
| 28 | "keyboard-configuration/variant=USA console-setup/ask_detect=false ", |
| 29 | "initrd=/install/initrd.gz -- <enter>" |
| 30 | ], |
| 31 | "output_directory": "virtualbox", |
| 32 | "vboxmanage": [ |
| 33 | ["modifyvm", "{{ .Name }}", "--memory", "4096"], |
| 34 | ["modifyvm", "{{ .Name }}", "--cpus", "2"] |
| 35 | ] |
| 36 | } |
| 37 | ], |
| 38 | "provisioners": [ |
| 39 | { |
| 40 | "type": "shell", |
| 41 | "scripts": [ |
| 42 | "provisioners/common/preflight.sh" |
| 43 | … more provisioners … |
| 44 | ], |
| 45 | "pause_before": "10s", |
| 46 | "override": { |
| 47 | "virtualbox-iso": { |
| 48 | "execute_command": "echo 'ubuntu' \| sudo -S bash {{.Path}}" |
| 49 | } |
| 50 | } |
| 51 | } |
| 52 | ] |
| 53 | } |
